# Supplementary material for: Elaborate cellulosome architecture of Acetivibrio cellulolyticus revealed by selective screening of cohesin–dockerin interactions
Source: PeerJ. 2014 Oct 30;2:e636. doi: 10.7717/peerj.636 (PMC4217186; doi:10.7717/peerj.636)
Supplement: Supplemental Information 1 [file peerj-02-636-s001.docx]

**Elaborate cellulosome architecture of *Acetivibrio cellulolyticus* revealed by selective screening of cohesin-dockerin interactions**

Yuval Hamberg^a^, Vered Ruimy-Israeli^a^, Bareket Dassa^a^, Yoav Barak^a,b^, Raphael Lamed^c^, Kate Cameron^d^, Carlos M. G. A. Fontes^d^, Edward A. Bayer^a*^ and Daniel B. Fried^a*^

**SUPPLEMENTAL FILES**

**SI Table 1A. Primer names and sequences for amplification of cohesin genes.** Forward (fw) and reverse (rv) primers are indicated for each of the cohesin genes examined in this study.

| **Primer sequence** | **Primer name** |
| --- | --- |
| 5'-ACATATGGATCCGTAATTGGTACTGGATTCACAGTTAGTG-3' | CohA3-fw |
| 5'-ATCTATCTCGAGCTATGTTGATATTGAACCACCAT-3' | CohA3-rv |
| 5'-ACATATGGATCCACAAAAACATCAACACCAAATCCT-3' | CohA4-fw |
| 5'-ATCTATCTCGAGCTAGATCTCTACTTTACCATCTG-3' | CohA4-rv |
| 5'-ACATATGGATCCATAGATGGAAAAGCTATGAAAG-3' | CohA5-fw |
| 5'-ATCTATCTCGAGCTAGTTTCCTACTGTAACAGAAC-3' | CohA5-rv |
| 5'-ACATATGGATCCACATCTACAGGTGATAGCTA-3' | CohB4-fw |
| 5'-ATCTATCTCGAGCTATGTTACACTTCTAACAGTTA-3' | CohB4-rv |
| 5'-ATATATGGATCCTCTGATTTACAGGTTGACATTG-3' | CohC3-fw |
| 5'-ATATATCTCGAGCTAACTTGCAATTACCTCAATTT-3' | CohC3-rv |
| 5'-CATGCAGGATCCGCAAGCGATGAGTCTTATATAACAATGGAT-3' | Coh-D1 fw |
| 5'-GTATGAAATAAAACAACCTTCAGATATTAAGGTTAAAGTATAGCTCGAGCACCAC-3' | Coh-D1 rv |
| 5'-ACATATGGATCCAATGGATTCCAAGTAAAAATTGGTTCGTATGTAGC-3' | CohD3-fw |
| 5'-ATCTATCTCGAGCTAAGTTACAGTCACTCCACTAT-3' | CohD3-rv |
| 5'-ATATATGGATCCACGCAAATGCCTTCAAATTA-3' | Coh-E7-fw |
| 5'-ATATATCTCGAGCTAGTTTATCTGAGCTGGTTGAA-3' | Coh-E7-rv |
| 5'-ATATATGGATCCGATTCAACCTCAAGTGTCAAAATT-3' | CohF1-fw |
| 5'-ATATATCTCGAGCTAGGTAGAAGTTACTGATGTTC-3' | CohF1-rv |
| 5'-ACATATGGATCCGCTGCAGATACTATTACTGT-3' | CohG-fw |
| 5'-ATCTATCTCGAGCTATACTTAAGGCATCTTTTGAT-3' | CohG-rv |
| 5'-ACATATGGATCCGCAGCTTCAAGTACTGAAACCGATACAAAC-3' | CohH-fw |
| 5'-ATCTATCTCGAGCTAATTAACACTGCCGGGATAGA-3' | CohH-rv |
| 5'-ACATATGGATCCGAATCTTCTATGCAGGTAAA-3' | CohJ-fw |
| 5'-ATCTATCTCGAGCTACGTTGCATCTCCAACTATTA-3' | CohJ-rv |
| 5'-ATATATGGATCCGGCATTAGCGAGACGGTCAATGTATC-3' | Coh-K2-fw |
| 5'-ATATATCTCGAGCTAAACAGTAATTTTTCCAATGC-3' | Coh-K2-rv |
| 5'-ACATATGGATCCGCAACTGGATTTACAGTTAGTGTAGGTT-3' | CohM1-fw |
| 5'-ATCTATCTCGAGCTATGTTGATACTGTTCCACCAC-3' | CohM1-rv |
| 5'-ACATATGGATCCGGAAGTGGATTTACAGTAAG-3' | CohM2-fw |
| 5'-ATCTATCTCGAGCTATGTTGATACTGTTCCACCAC-3' | CohM2-rv |
| 5'-ATATATGGATCCGGAACTGGATTCACTGTTAG-3' | CohM3-fw |
| 5'-ATATATCTCGAGCTATGTTGATACTGTTCCTCCAC-3' | CohM3-rv |
| 5'-ACATATGGATCCAGCACTGCTGCCTCATCTATGTAT-3' | CohN-fw |
| 5'-ATCTATCTCGAGCTATGAATCTTGAGAATTAACCA-3' | CohN-rv |
| 5'-ACATATGGATCCAATAGTGCAATAAATATTTC-3' | CohO-fw |
| 5'-ATCTATCTCGAGCTAAGCCGATGTCTGCAAACTAC-3' | CohO-rv |
| 5'-ACATATGGATCCGCAGGAATAGTCAAGATGGATTTT-3' | Coh-P-fw |
| 5'-ATCTATCTCGAGCTATAAGCTGCTATTTACAATCT-3' | Coh-P-rv |
| 5'-ATATATGGATCCTCTTCATTCGAGATTACTGTG-3' | Coh-ScaL3-fw |
| 5'-ATATATCTCGAGCTAAGATTGAGGGTTTGGTGTTT-3' | Coh-ScaL3-rv |

### SI Table 1B. Primer names and sequences for amplification of dockerin genes. Forward (fw) and reverse (rv) primers are indicated for each of the dockerin genes examined in this study.

| **Primer sequence** | **Primer name** |
| --- | --- |
| 5'-ACATATGGTACCAGCTGGGATTGCCATGCCTGAAGG-3' | Doc_Doc-fw |
| 5'-ATCTATGGATCCCTATTAATTTAATATTGGACCTG-3' | Doc_Doc-rv |
| 5'-ACATATGGTACCAAAATTTATATATGGTGATGT-3' | DocB-fw |
| 5'-ATCTATGGATCCCTAATGCCTAGATTATTCTTCTT-3' | DocB-rv |
| 5'-ACATATGGTACCAAGCAGTAATACAAATACTGTATCAGGATATATCAAGTCCG-3' | Doc-Xyn10-CBMs-fw |
| 5'-ATCTATGGATCCCTATTTGTAACTTGCAGAAGTTT-3' | Doc-Xyn10-CBMs-rv |
| 5'-ACATATGGTACCAGCTGTTACGGGGGATATTAA-3' | DocCel124-fw |
| 5'-ATCTATGGATCCCTAAGTTTGAGTTCCTCCATTTG-3' | DocCel124-rv |
| 5'-ACATATGGTACCAACCCCTACTGGATTGAAGGGTGACGTAGATCTT-3' | DocCel48-fw |
| 5'-ATCTATGGATCCCTATTAATTTTGTGAGAGTAATT-3' | DocCel48-rv |
| 5'-ACATATGGTACCAGCACAATACGTATATGGTGA-3' | Doc-Cel9-ZP_09464491-fw |
| 5'-ATCTATGGATCCCTACTTTTGTACCGGAAACTTTG-3' | Doc-Cel9-ZP_09464491-rv |
| 5'-ACATATGGTACCAGCAGGAAAAGTTGATAGTAT-3' | DocK1-fw |
| 5'-ATCTATGGATCCCTAAACTTCCTCATCAATAGGCT-3' | DocK1-rv |
| 5'-ACATATGGTACCATCACCAATGATACTTGGAGA-3' | DocK2-fw |
| 5'-ATCTATGGATCCCTAACCTTCATTTGATTCTGTTG-3' | DocK2-rv |
| 5'-ACATATGGTACCAGATGGAACATCAGCATCATCAAC-3' | DocO-fw |
| 5'-ATCTATGGATCCCTATGTCATGGTATCTTTAGGAA-3' | DocO-rv |
| 5'-ACATATGGTACCAGATAGTACTCTATTTAAGGAAAGTGAAAAGTTTGATGACCC-3' | Doc-tri-ZP_09463877-fw |
| 5'-ATCTATGGATCCCTAAAACTGTGTTATTTCATAGC-3' | Doc-tri-ZP_09463877-rv |
| 5'-ACATATGGTACCAAACATTAGTACTATATTAGA-3' | Doc-tri-ZP_09464147-fw |
| 5'-ATCTATGGATCCCTACTATTTATCTGAATAGTAAG-3' | Doc-tri-ZP_09464147-rv |
| 5'-ACATATGGTACCAACAGCGCCGCCTCCTGAAATTCT-3' | Doc-ZP_09463099-fw |
| 5'-ATCTATGGATCCCTATATGCACAAAATCCATTTTC-3' | Doc-ZP_09463099-rv |
| 5'-ACATATGGTACCAAAGGAAGAAGCGAGCAATGCTCTA-3' | Doc-ZP_09465673-fw |
| 5'-ATCTATGGATCCCTAAATCTGATTTACAACTGGAA-3' | Doc-ZP_09465673-rv |
| 5'-CGAAGTGGTACCTATTGGTATAGTATCTGAAGGAACTACAGTTTCAGGC-3' | XDoc-A fw |
| 5'-GCAGCCGGATCCTTAATAGTCTGAAGATACTTTATTGAAGTGTTTAGCAA-3' | XDoc-A rv |
| 5'-ACATATGGTACCAGGCAGTGTTAATGTTTTTGCTGAGCCTGAATACAACAC-3' | X'DocH-fw |
| 5'-ATCTATGGATCCCTAGCTACTTATATTTACGGAGT-3' | X'DocH-rv |
| 5'-ACATATGGTACCAGCGAATTCACAAGCTGTTGGAAGCAG-3' | XDocP-fw |
| 5'-ATCTATGGATCCCTAATAATCCTCAGGAAATTTAA-3' | XDocP-rv |
| 5'-ACATATGGTACCAGCTGGGATTGCCATGCCTGAAGG-3' | ZP_09465996-DocDoc-fw |
| 5'-ATCTATGGATCCCTATGTTTAATTTAATATTGGAC-3' | ZP_09465996-DocDoc-rv |
